# Multi-Parameter Characterization of HEMA/BPA-free 1- and 2-step Universal Adhesives Bonded to Dentin

**DOI:** 10.3290/j.jad.b4949669

**Published:** 2024-02-08

**Authors:** Chuliang Tang, Mohammed H. Ahmed, Kumiko Yoshihara, Marleen Peumans, Bart Van Meerbeek

**Affiliations:** a PhD Student, KU Leuven (University of Leuven), Department of Oral Health Sciences, BIOMAT – Biomaterials Research group & UZ Leuven (University Hospitals Leuven), Dentistry, Leuven, Belgium. Performed the experiment and statistical analysis, wrote the manuscript.; b Junior Postdoctoral Researcher, KU Leuven (University of Leuven), Department of Oral Health Sciences, BIOMAT – Biomaterials Research Group & UZ Leuven (University Hospitals Leuven), Dentistry, Leuven, Belgium; Tanta University, Faculty of Dentistry, Department of Dental Biomaterials, Tanta, Egypt. Performed the experiment and statistical analysis, proofread the manuscript.; c Senior Researcher, National Institute of Advanced Industrial Science and Technology (AIST), Health and Medical Research Institute, Kagawa, Japan; Okayama University, Graduate School of Medicine, Dentistry and Pharmaceutical Sciences, Department of Pathology & Experimental Medicine, Okayama, Japan. Contributed substantially to discussion, proofread the manuscript.; d Professor, KU Leuven (University of Leuven), Department of Oral Health Sciences, BIOMAT – Biomaterials Research group & UZ Leuven (University Hospitals Leuven), Dentistry, Leuven, Belgium. Contributed substantially to discussion, proofread the manuscript.; e Full Professor, KU Leuven (University of Leuven), Department of Oral Health Sciences, BIOMAT – Biomaterials Research group & UZ Leuven (University Hospitals Leuven), Dentistry, Leuven, Belgium. Idea, experimental design, proofread the manuscript, contributed substantially to discussion.; * Equal first-author contribution.

**Keywords:** dental bonding, bond durability, water sorption, interface, HEMA, BPA

## Abstract

**Purpose:**

This study aimed to investigate the bonding effectiveness of two HEMA/BPA-free universal adhesives (UAs) to flat dentin, to characterize their adhesive-dentin interfacial ultrastructure, and to measure their water sorption (Wsp), water solubility (Wsl), and hydrophobicity.

**Materials and Methods:**

The immediate and aged (50,000 thermocycles) microtensile bond strength (μTBS) to flat dentin of the HEMA/BPA-free UAs Healbond Max (HbMax; Elsodent) and Healbond MP (HbMP; Elsodent) as well as the reference adhesives OptiBond FL (Opti-FL; Kerr), Clearfil SE Bond 2 (C-SE2; Kuraray Noritake), and Scotchbond Universal (SBU; 3M Oral Care) was measured. The adhesive-dentin interfaces of HbMax and HbMP were characterized by TEM. Wsp and Wsl of all adhesive resins and of the primer/adhesive resin mixtures of HbMax, Opti-FL, and C-SE2 were measured. Hydrophobicity was determined by measuring the contact angle of water dropped on adhesive-treated dentin.

**Results:**

In terms of µTBS, HbMax and HbMP performed statistically similarly to Opti-FL and C-SE2, but outperformed SBU. Aging only significantly reduced the μTBS of SBU when applied in E&R bonding mode. TEM revealed typical E&R and SE hybrid-layer ultrastructures at dentin, while electron-lucent globules of unknown origin, differing in size and shape, were observed within the adhesive resin of HbMP and even more frequently in that of HbMax. Higher Wsp was measured for the primer/adhesive resin mixtures than for the adhesive resins. Opti-FL was more hydrophobic than all other adhesives tested.

**Conclusion:**

The HEMA/BPA-free UAs bonded durably to flat dentin with bond strengths comparable to those of the gold-standard E&R/SE adhesives and superior to that of the HEMA/BPA-containing 1-step UA.

Since Buonocore first attempted in 1955 to bond to tooth tissue using the acid-etch technique,^5^ dental adhesives have gradually and continuously evolved in composition, number/kind of application steps, and adhesive approaches. While stable and satisfactory long-term adhesion to enamel can relatively easily be achieved via the acid-etch technique, bonding to dentin through hybridization has always been more challenging. Contrasting hydroxyapatite (HAp)-free and HAp-rich hybrid layers are formed on dentin when bonding is performed either using an etch-and-rinse (E&R) or self-etch (SE) adhesive approach, respectively. The most important difference is that E&R adhesives involve a separate phosphoric-acid etching step, while SE adhesives make use of acidic functional monomers, by which water rinsing is no longer needed.^49^ Although traditional and in particular multi-step/bottle E&R and SE dental adhesives have more often been documented with superior long-term clinical performance, clinical findings are not always consistent, as the bonding performance of dental adhesives also depends on non-material parameters such as operator/clinician- and patient-related factors.^37,47^

Offering three options of application in either a full E&R, full SE, or combined E&R-on-enamel/SE-on-dentin bonding mode, universal adhesives (UAs) comprise the most recent generation of adhesives. They follow the trend of simplified and fast bonding by blending hydrophobic and hydrophilic components into one-bottle adhesive solutions. As major limitations, 1-step UAs (1-UAs) are usually applied in a film thinner than 10 μm and exhibit relatively high hydrophilicity. This makes them susceptible to reduced polymerization and enhanced hydrolytic degradation, obviously lowering their bonding performance; this concern has been raised before for simplified 2-step E&R and 1-step SE adhesives.^8^ To compensate for the shortcomings of 1-UAs, 2-step/bottle UAs (2-UAs) have more recently been introduced.^41,51^

To decrease the hydrophilicity of dental adhesives, one attempt is to omit the addition of 2-hydroxyethyl methacrylate (HEMA). Being a hydrophilic mono-functional monomer with a low molecular weight, HEMA is still frequently incorporated into adhesives, including UAs, in order to act as co-solvent for other monomer ingredients and promote resin infiltration into the fully E&R or partially SE demineralized dentin.^4,27^ On the other hand, the high hydrophilic nature of HEMA due to its hydroxyl group may harm bond longevity, because HEMA retains water at the adhesive-dentin interface, does not polymerize efficiently, promotes water uptake through osmosis from (vital) dentin or the wet outer oral environment.^1,26,46,48^ It also impairs bonding of SE adhesives by inhibiting the interaction of 10-MDP with Ca.^6,52^ Other drawbacks are the allergenic and cytotoxic side-effects of HEMA.^20,39,46^

Apart from bonding efficacy, concerns regarding the biosafety/compatibility of dental materials are rising. In addition to potential adverse effects of HEMA, more attention is also paid to bisphenol A (BPA) and its potential release from resin-based dental materials like adhesives.^11^ BPA is an endocrine disruptor inducing estrogenic effects, as it can bind to estrogen receptors.^35^ BPA has also been documented to affect several organs and physiological systems, causing adverse reproductive and developmental side-effects.^50^ Although BPA is not intended to be incorporated into dental adhesives, it may remain as impurity from synthesis, and may also be produced by degradation of bisphenol A-glycidyl methacrylate (bis-GMA).^11^ Bis-GMA is one of the most frequently used cross-linking monomers in adhesives and other resin-based dental materials.^45^ Demands for BPA-alternatives and BPA-free materials are rising, due to the demand to reduce daily BPA intake.^15^

In this study, the HEMA- and BPA-free 2-UA Healbond Max (Elsodent; Cergy-Pontoise, France) and its simplified 1-UA version Healbond MP (Elsodent) were investigated for multiple parameters, such as bonding effectiveness immediately and upon aging, interfacial interaction with dentin, water sorption and solubility, as well as hydrophobicity. The null hypotheses tested in this study were that (1) the bond strength of the HEMA/BPA-free UAs to dentin, (2) their water sorption and solubility, and (3) hydrophobicity would not differ from those of the reference 3-E&R, 2-SE, and 1-UA adhesives.

## Materials and Methods

### Microtensile Bond Strength (μTBS) to Flat Dentin

Forty non-carious human third molars were collected following informed consent, as approved by the Commission of Medical Ethics of the KU Leuven (University of Leuven), file number S64350. The collected teeth were stored in an aqueous solution of 0.5% chloramine-T trihydrate at 4°C and used within 2 months. Subsequently, the teeth were randomly divided into five experimental groups consisting of 8 teeth per experimental group.

Before specimen preparation, the teeth were warmed up in 100% humidity to 37°C for at least 30 min. The occlusal third of the crowns was removed using a water-cooled slow-speed diamond saw (IsoMet, Buehler; Lake Bluff, IL, USA) after having mounted the teeth in gypsum blocks. Next, a uniform, standardized, and clinically representative bur-cut smear layer was prepared on all exposed mid-coronal dentin surfaces using a high-speed medium-grit (107-µm) diamond bur (882, Komet; Lemgo, Germany) mounted in a customized Micro-Specimen Former (University of Iowa, Iowa City, IA, USA). The prepared dentin surfaces were examined for absence of enamel and/or exposure of pulp tissue using a stereomicroscope (Wild M5A, Wild Heerbrugg; Heerbrugg, Switzerland). A split-tooth design was applied for three experimental groups testing 2-UA Healbond Max (HbMax; Elsodent), 1-UAs Healbond MP (HbMP; Elsodent), and Scotchbond Universal (SBU; 3M Oral Care; Seefeld, Germany), with these UAs applied in E&R mode on one tooth half and in SE mode on the other corresponding tooth half. The reference E&R adhesive OptiBond FL (Opti-FL, Kerr; Orange, CA, USA) and the reference SE adhesive (C-SE2, Kuraray Noritake; Tokyo, Japan) were applied to the whole dentin surface in solely E&R or SE bonding mode, respectively. Following the split-tooth design (UAs only), the flat dentin surface was divided into two nearly equal parts by cutting a shallow 1-mm deep groove in the center using a thin 150-μm diamond blade (IsoMet, Buehler). Prior to phosphoric-acid etching, a single-edge carbon-steel blade (Electron Microscopy Sciences; Hatfield, PA, USA) was positioned in the groove between the two tooth halves in order to prevent acid leakage from the E&R half to the SE half. Each prepared tooth was immediately transferred to the 37°C incubator at 100% humidity awaiting the application of the adhesive. All dental adhesives tested in this study were applied according to their respective manufacturer’s instructions for use (Table 1). Upon bonding, each tooth was restored in four 1.5-mm increments of composite (Clearfil AP-X, Kuraray Noritake; shade A3) to achieve a 5-6 mm build-up height. Each increment was light cured for 10 s, with each side of the completed composite build-up additionally light cured for 10 s, thus ensuring sufficient total curing time and making it impossible that insufficient light curing could have affected the data. All light curing done in this study was carried out using the LED light-curing unit Bluephase 20i (Ivoclar; Schaan, Liechtenstein) in “high” mode with a power output of at least 1200 mW/cm^2^, as confirmed by a Marc Resin Calibrator (BlueLight Analytics; Halifax, Canada). Following light curing, the restored teeth were stored for 24 h in the incubator at 37°C under 100% humidity, then transferred into pre-warmed distilled water at 37°C and stored for 6 days.

**Table 1:** List of the experimental and reference/control dental adhesives investigated in this study

Adhesive	Composition	Application
HealBond Max (HbMax, Elsodent)	Primer: 10-MDP, hydrophilic dimethacrylate, photo-initiator, ethanol, water Bond: 10-MDP, hydrophilic and hydrophobic dimethacrylates, modified urethane dimethacrylate, dl-camphorquinone, silanated colloidal silica, initiators, accelerators	E&R: Etch for 15 s using Gel Etchant (Kerr), thoroughly rinse with water (>10 s), gently air dry, then proceed as for SE. SE: Apply primer in rubbing motion for 20 s, gently air dry (≥10 s) until the primer no longer moves, apply Bond with brushing motion for 15 s, gently air blow to make a uniform layer (5 s) and light cure for 10 s.
HealBond MP (HbMP, Elsodent)	10-MDP, silane, hydrophilic and hydrophobic dimethacrylate, silanated colloidal silica, ethanol, water, dl-camphorquinone, initiators, accelerators	E&R: Etch for 15 s using Gel Etchant, thoroughly rinse with water (>10 s), gently air dry, then proceed as for SE. SE: Apply adhesive in rubbing motion for 15 s, gently air dry (≥10 s) until the adhesive does no longer move and light cure for 10 s.
Scotchbond Universal (SBU, 3M Oral Care)	10-MDP, HEMA, silane, dimethacrylates, Vitrebond copolymer, filler, ethanol, water, initiators	E&R: Etch for 15 s using Gel Etchant, thoroughly rinse with water (>10 s), gently air dry, then proceed as for SE. SE: Apply adhesive, rub the surface for 20 s, gently air dry for 5 s until the adhesive does no longer move and light cure for 10 s.
OptiBond FL (Opti-FL, Kerr)	Primer: GPDM, HEMA, 2-[2-(methacryl-oyloxy)ethoxycarbonyl] benzoic acid, ethanol, water. Adhesive (Bond): barium-aluminum borosilicate glass, fumed silica, HEMA, ytterbium trifluoride, trimethoxy-silylpropyl methacrylate, 2-hydroxy-1,3-propanediyl, bismethacrylate, disodium hexafluorosilicate	E&R: Etch for 15 s using Gel Etchant, thoroughly rinse with water (>10 s), gently air dry, apply Primer with a light scrubbing motion for 15 s, gently air dry for 5 s, apply adhesive, gently air dry to make a uniform layer and light cure for 10 s.
Clearfil SE Bond 2 (C-SE2, Kuraray Noritake)	Primer: 10-MDP, HEMA, hydrophilic dimethacrylate, photo-initiator, water Bond: 10-MDP, HEMA, bis-GMA, hydrophobic dimethacrylate, dl-camphorquinone, silanated colloidal silica, initiators, accelerators	SE: Apply primer using a microbrush, leave undisturbed for 20 s, gently air dry until the primer no longer moves (>5 s), apply Bond, gently air dry to make a uniform layer and light cure for 10 s.
Gel Etchant (Kerr)	Phosphoric acid (37.5%)	Etch for 15 s, thoroughly rinse with water (>10 s) and gently air dry

10-MDP: 10-methacryloyloxydecyl dihydrogen phosphate; bis-GMA: bisphenol A-glycidyl methacrylate; HEMA: 2-hydroxyethyl methacrylate; GPDM: glycero-phosphate dimethacrylate.

After 1-week storage, as mentioned above, the teeth were sectioned using a precision-cutting machine (Accutom 50, Struers; Ballerup, Denmark) to produce 1-mm^2^ (±0.1 mm) sticks. The 6 central microspecimens originating from the central tooth area were collected from each tooth half (12 microspecimens in total from each tooth; 6 microspecimens/bonding mode). The bonded interfaces of all microspecimens were examined for absence of enamel using the stereomicroscope. Half of the microspecimens were measured immediately (no thermocycling) and referred to as immediate “0TC μTBS”. The remaining half was subjected to 50,000 thermocycles using the thermocycler THE-1200 (SD Mechatronik; Munich, Germany), and referred to as aged “50kTC μTBS”. The exact microspecimen dimensions were measured using a digital caliper (DIGI-MET, Helios Preisser; Gammertingen, Germany), prior to fixing the specimens to customized jigs with cyanoacrylate glue (Model Repair II Blue, Dentsply-Sankin; Ohtawara, Japan). The non-trimmed rectangular microspecimens were stressed in tension using a universal testing device (LRX, Lloyd; Hampshire, UK) at a crosshead speed of 1 mm/min, upon which the μTBS was calculated in MPa by dividing the force imposed at the time of fracture in N by the bond area (mm^2^). Any pre-test failures (ptfs) during cutting, storage, aging, and/or jig mounting were explicitly recorded and included as 0 MPa. The μTBS test guidelines prescribed by the Academy of Dental Materials were strictly followed.^2^ All fractured μTBS microspecimen pairs (dentin and composite side) were examined using stereomicroscopy (Wild M5A, Wild Heerbrugg) to determine the mode of failure, which was recorded either as cohesive in dentin (dentin), adhesive interfacial (interface), mixed (mixed), or cohesive in composite (composite). Three μ-specimens, representative of each experimental group, were prepared for scanning electron microscopy following a conventional process, including fixation in 2.5% glutaraldehyde in 0.1 M sodium cacodylate buffer, rinsing with 0.2 M sodium cacodylate buffer, gradual dehydration in ethanol, and drying using hexamethyldisilazane (HMDS). Upon gold-sputter coating (JFC-1300, JEOL; Tokyo, Japan), the specimens were examined using a scanning electron microscope (SEM, JSM-6610LV, JEOL).

### TEM Characterization of the Adhesive-Dentin Interfacial Ultrastructure

Flat dentin surfaces were prepared according to the procedure described above (n=2/experimental group). The dental adhesives HbMax and HbMP were applied both in E&R and SE mode, then light cured for 10 s before the application of one approximately 1-mm-thick layer of flowable composite (G-ænial Universal Flo, GC; Tokyo, Japan). After being stored in 100% humidity for 24 h and distilled water at 37°C for 6 days, the specimens were sectioned into 0.6- to 0.8-mm-thick slabs.

Both non-demineralized and demineralized specimens were processed for TEM according to routine TEM-specimen processing, as described in detail before, including immersion in 2% formaldehyde-formic acid solution (Gooding & Stewart fluid, Prosan; Gent, Belgium) for 38 h (only for demineralized specimens), fixation in 2.5% glutaraldehyde in 0.1 M sodium cacodylate buffer for at least 12 h, rinsing with 0.1 M sodium cacodylate buffer for 1 min with 3 changes, dehydration in ascending concentrations of ethanol solutions (25%, 50%, 75%, 95%, and 100%) for 10 min and 2 times each, immersion in 99% propylene oxide for 10 min and 3 times, and finally embedding in epoxy resin (Epoxy embedding medium, Sigma-Aldrich; St Louis, MO, USA).

Ultrathin sections (70-90 nm) were prepared using an ultramicrotome (Ultracut UCT, Leica; Vienna, Austria) equipped with a 45-degree TEM diamond knife (Diatome; Nidau, Switzerland) prior to being examined with TEM (JEM-1400 Flash, JEOL), unstained (for non-demineralized sections) or positively stained (for demineralized sections) with UranyLess (Electron Microscopy Sciences; Hatfield, PA, USA) for 8 min and lead citrate (Electron Microscopy Sciences) for 3 min.

### Water Sorption (Wsp) and Water Solubility (Wsl)

Adhesive-resin disks (n=15) of 1-UAs SBU and HbMP, the pure adhesive resin of HbMax, Opti-FL, and C-SE2, and a 1:3 weight-ratio mixture of the respective primer and adhesive resin of HbMax, Opti-FL, and C-SE2 were prepared using silicone molds (15 mm diameter, 1 mm thickness). The adhesive disks were light cured for 60 s using the LED light-curing unit Bluephase 20i (Ivoclar), used in “high” mode with a light output of at least 1200 mW/cm^2^ (see above), from each side to guarantee optimum polymerization. Each disk was polished with P600 and P1200 SiC papers (WS Flex 18C, Hermes Schleifmittel; Hamburg, Germany), after which its height and diameter were measured using a digital caliper (Holex, Hoffmann Group; Munich, Germany) to calculate the disk volume (V). Next, the disks were dried using the same method as described before, including storage at 37°C for 22 h in an incubator (Heratherm, Thermo Fisher Scientific; Waltham, MA, USA) and at 23°C room temperature for 2 h. Then they were transferred to a glass desiccator and weighed every 24 h using a calibrated electronic analytical balance (AB304-S Analytical Balance, Mettler Toledo; Greifensee, Switzerland) to reach a constant mass, which was recorded as m1. Each disk was next immersed in 10 ml distilled water and stored in the incubator at 37°C. The disks of the same group were divided into three subgroups and stored for 1 week, 6 months, or 1 year, with 5 specimens for each period. The weight of each disk was measured after water storage and recorded as m2. Then, the disks were again dried in the glass desiccator to reach a constant mass, being referred to as m3. Water sorption (Wsp) and water solubility (Wsl) in μg/mm^3^ were calculated according to formulae 1 and 2, respectively:


$$\begin{array}{llll} \text{Wsp} & = & \frac{m2-m3}{\nu } & \text{formula }1\\ \text{Wsl} & = & \frac{m1-m3}{\nu } & \text{formula }2 \end{array}$$

### Contact Angle (CA) Measurement of Hydrophobicity/Hydrophilicity

Flat dentin surfaces were prepared according to the procedure described above (n=3/experimental group). Adhesives were applied according to the manufacturer’s instructions (Opti-FL in E&R bonding mode, others in SE bonding mode) and light cured for 10 s. The contact angle of a drop of Milli-Q water on adhesive-treated dentin surfaces was measured using an optical contact-angle measuring and contour-analysis system (OCA 15EC, Dataphysics; Filderstadt, Germany).

### Statistical Analysis

A linear mixed-effects model (LME, R Foundation for Statistical Computing; Vienna, Austria) was applied to analyze the μTBS recorded for the adhesives investigated, with the significance level set at α = 0.05. Three variables – adhesive, aging, and bonding mode – were included as fixed factors and the variable “tooth” was listed as the random factor. The non-parametric Kruskal-Wallis test with a significance level of α = 0.05 was carried out using the same software to statistically analyze Wsp and Wsl, followed by post-hoc multiple comparisons with Bonferroni adjustment. CA was statistically analyzed using one-way ANOVA, followed by the post-hoc Tukey test.

## Results

### μTBS to Flat Dentin and Failure Analysis

**Fig 1:**
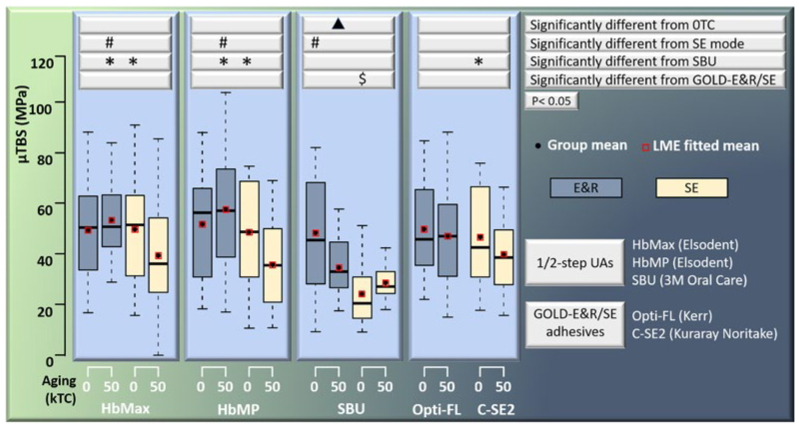
Box-and-whisker plots of the 0TC and 50kTC aged μTBS of adhesives bonded to flat dentin applied in E&R and/or SE bonding modes. The horizontal lines from the top to the bottom for each box represent the maximum, the upper quartile, the median, the lower quartile and the minimum value of the group (excluding the outliers). Statistical differences in μTBS among the different experimental groups are indicated by specific symbols for specific parameters (p < 0.05).

All μTBS data are detailed in Table 2 and graphically presented in boxplots in Fig 1. The third interaction – adhesive x bonding mode x aging – significantly contributed to the LME model (Table 3), revealing that the combination of any two parameters varied for the different levels of the third parameter. When compared to the gold-standard E&R (Opti-FL) and SE (C-SE2) adhesives, only the immediate 0TC μTBS of the 1-UA SBU applied in SE bonding mode was significantly lower. The immediate 0TC μTBS of HbMax and HbMP, when applied in SE bonding mode, and the aged 50kTC μTBS of HbMax and HbMP, when applied in E&R bonding mode, were significantly higher than the respective μTBS of SBU. A significant difference was only found between the immediate 0TC and aged 50kTC μTBS for SBU when applied in E&R bonding mode. Comparing the bonding modes, the immediate 0TC μTBS of SBU and the aged 50kTC μTBS of HbMax and HbMP were significantly higher when the adhesives were applied in E&R than in SE bonding mode.

**Table 2:** Immediate and aged μTBS of the adhesives investigated to flat dentin

μTBS (MPa)*	0TC		50kTC	
	E&R	SE	E&R	SE
HbMax	49.3 ± 18.8 (0/24)	49.7 ± 21.6 (0/24)	53.4 ± 15.1 (0/24)	39.3 ± 20.5 (1/24)
HbMP	51.7 ± 20.6 (0/23)	48.5 ± 20.0 (0/23)	57.6 ± 24.6 (0/23)	35.7 ± 18.3 (0/23)
SBU	48.3 ± 21.3 (0/24)	24.1 ± 12.3 (0/24)	34.5 ± 10.9 (0/24)	28.6 ± 6.7 (0/24)
C-SE2	–	46.6 ± 19.1 (0/24)	–	39.8 ± 14.0 (0/24)
Opti-FL	49.7 ± 18.3 (0/24)	–	47.0 ± 18.9 (0/24)	–

*Mean ± SD (ptf/n); SD: standard deviation; ptf: pre-test failure; n: number of micro-specimens.

**Table 3:** Statistical analysis of the fixed variables and interactions for the LME model

	numDF	denDF	F-value	p-value
(Intercept)	1	330	1089.1338	<0.0001*
Adhesive	3	36	5.9801	0.0020*
Bonding mode	1	330	31.6259	<0.0001*
Aging	1	330	5.1895	0.0234*
Adhesive x bonding mode	3	330	1.4730	0.2217
Adhesive x aging	3	330	0.0544	0.9833
Bonding mode x aging	1	330	1.7113	0.1917
Adhesive x bonding mode x aging	3	330	5.5002	0.0011*

*Statistically significant.

**Fig 2:**
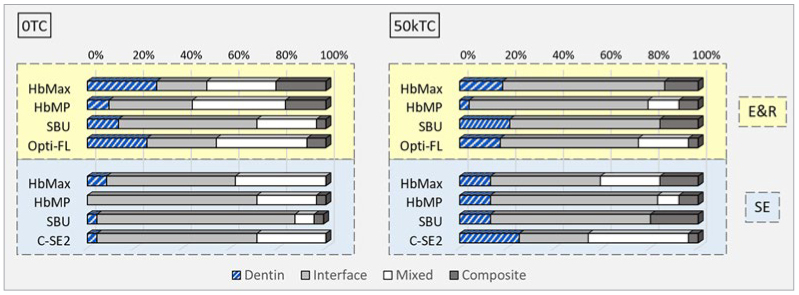
Light-microscopy failure analysis presenting the failure-mode distribution of fractured µ-specimens: dentin: cohesive failure in dentin; interface: adhesive interfacial failure; mixed: mixed failure; composite: cohesive failure in composite.

**Fig 3:**
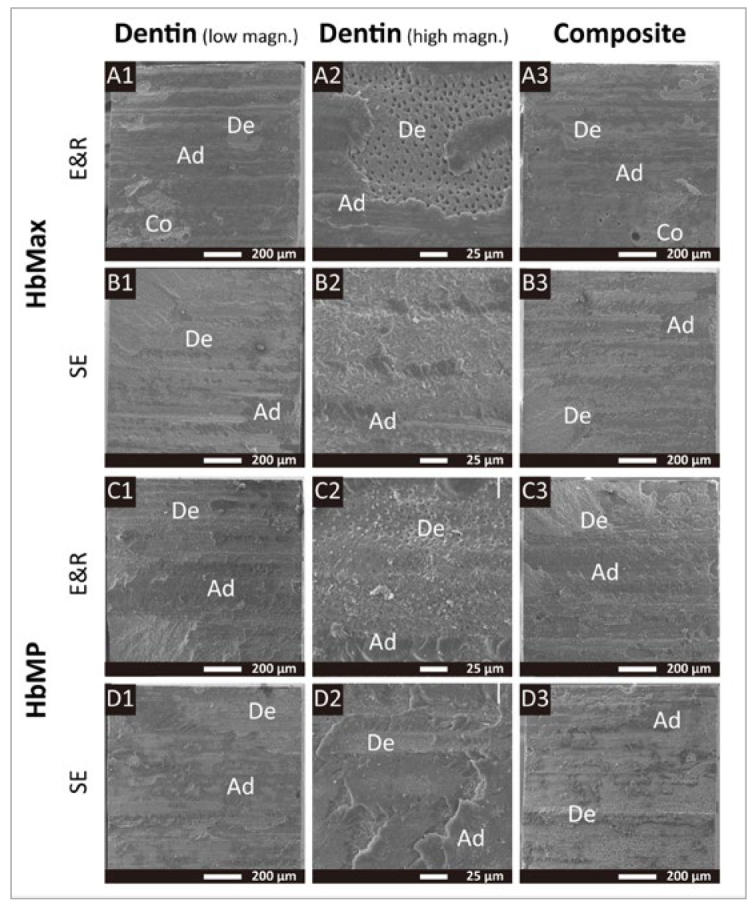
Representative SEM photomicrographs of fractured microspecimen surfaces of HbMax and HbMP applied in E&R and SE mode upon 50kTC aging. A1 to A3: Representative adhesive interfacial failure of HbMax_E&R visible on the dentin side in A1 with high magnification in A2, and visible on the composite side in A3. B1 to B3: Representative adhesive interfacial failure of HbMax_SE visible on the dentin side in B1 with high magnification in B2, and visible on the composite side in B3. C1 to C3: Representative mixed failure of HbMP_E&R visible on the dentin side in C1 with high magnification in C2, and viewed at the composite side in C3. D1 to D3: Representative mixed failure of HbMP_SE visible on the dentin side in D1 with high magnification in D2, and visible on the composite side in D3. Ad: adhesive; Co: composite; De: dentin.

**Fig 4:**
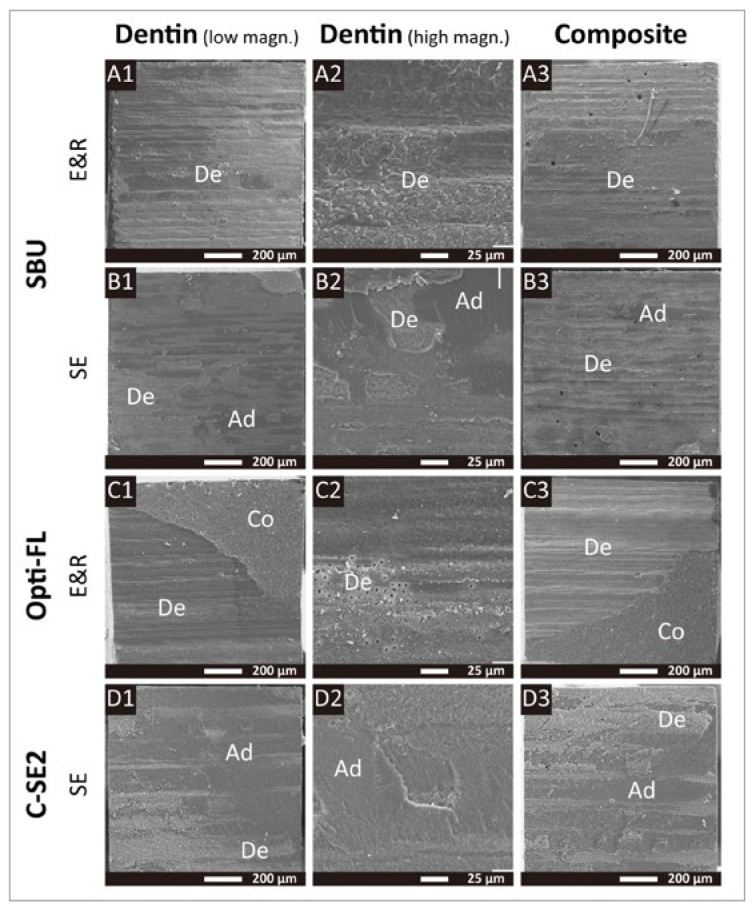
Representative SEM photomicrographs of fractured micro-specimen surfaces of SBU applied in E&R and SE mode, Opti-FL applied in E&R mode and C-SE2 applied in SE mode, all upon 50kTC aging. A1 to A3: Representative adhesive interfacial failure of SBU_E&R visible on the dentin side in A1 with high magnification in A2, and visible on the composite side in A3. B1 to B3: Representative adhesive interfacial failure of SBU_SE visible on the dentin side in B1 with high magnification in B2, and visible on the composite side in B3. C1 to C3: Representative mixed failure of Opti-FL_E&R visible on the dentin side in C1 with high magnification in C2, and visible on the composite side in C3. D1 to D3: Representative mixed failure of C-SE2_SE visible on the dentin side in D1 with high magnification in D2, and visible on the composite side in D3. Ad: adhesive; Co: composite; De: dentin.

The fracture analysis is graphically presented in Fig 2. SEM photomicrographs of fractured 50kTC-aged specimens representing the different dental adhesives investigated are shown in Figs 3 and 4. In general, the most frequently observed failure pattern was adhesive interfacial failure. Aging did not substantially change the distribution of failure patterns, except for a a slight but noticeable increase in the adhesive interfacial failure mode recorded for HbMax, HbMP, and Opti-FL upon 50kTC aging and when these adhesives were applied in E&R bonding mode.

### TEM Adhesive-Dentin Interfacial Characterization

**Fig 5:**
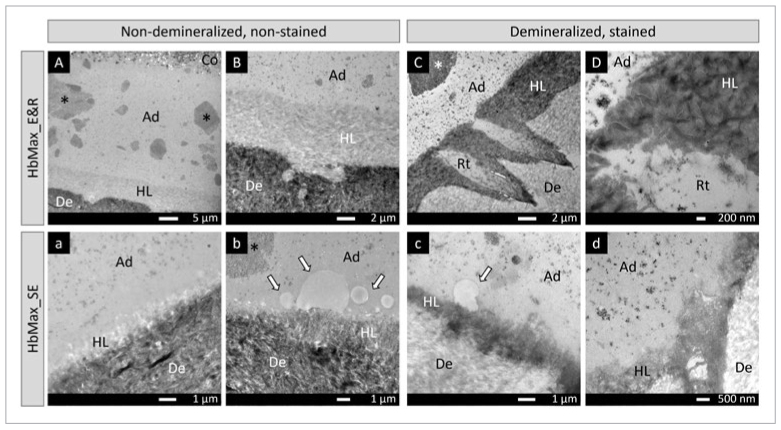
TEM photomicrographs representatively illustrating the ultra-structure of the adhesive-dentin interface of HbMax bonded to flat dentin following an E&R (A-D) and SE (a-d) bonding mode. A: Non-demineralized, non-stained section revealing a thick adhesive layer of about 30 µm. Within the adhesive layer, electron-dense features of different size and shape are visible, representing silica-filler agglomeration in several areas (asterisks). B: Higher magnification of (A), showing a fully demineralized E&R hybrid layer with a thickness of about 6 µm and an abrupt transition to unaffected dentin. C: Demineralized, stained section presenting a homogeneously and heavily stained E&R hybrid layer, while two distinct resin tags were formed within the opened dentinal tubules. The adhesive resin presents a relatively large silica-filler agglomeration somewhat remote from the hybrid layer (asterisk). D: Higher magnification of (C) showing cross-banded collagen fibrils within the E&R hybrid layer. a: Non-demineralized, non-stained section showing a partially demineralized SE hybrid layer. b: Non-demineralized, non-stained section revealing electron-lucent globules of different sizes immediately adjacent of the hybrid layer and of unknown origin (arrows). The adhesive resin also presents a relatively large silica-filler agglomeration somewhat remote from the hybrid layer (asterisk). c: Demineralized, stained section showing a homogeneously stained SE hybrid layer with a thickness of about 1 µm. A single globule with low electron density could be observed attached to the hybrid layer (arrow). d: Demineralized, stained section showing the SE hybrid layer. Ad: adhesive; Co: composite; De: dentin; HL: hybrid layer; Rt: resin tag.

**Fig 6:**
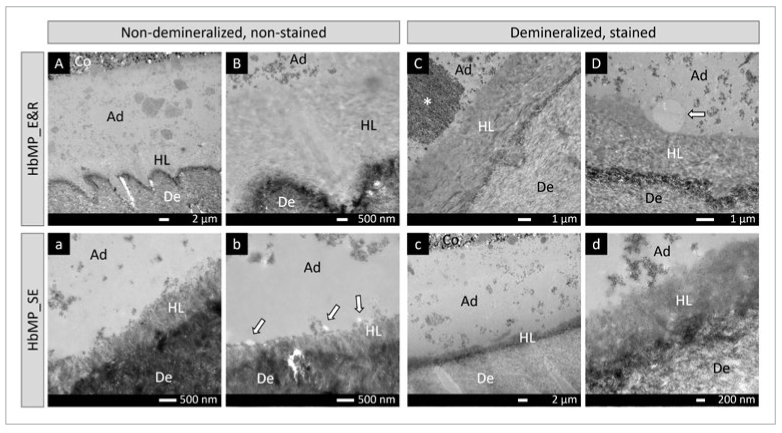
TEM photomicrographs representatively illustrating the ultra-structure the adhesive-dentin interface of HbMP bonded to flat dentin following an E&R (A-D) and SE (a-d) bonding mode. A: Non-demineralized, non-stained section revealing a thick adhesive layer of about 17 µm. B: Higher magnification of (A), showing a fully demineralized E&R hybrid layer with a thickness of about 5 µm. C: Demineralized, stained section presenting a homogeneously stained E&R hybrid layer. The adhesive resin presents a relatively large silica-filler agglomeration within the adhesive resin layer (asterisk). D: Demineralized, stained section showing an electron-lucent globule attached to the hybrid layer (arrow). a: Non-demineralized, non-stained section showing a partially demineralized SE hybrid layer with a thickness of about 0.8 µm. b: Non-demineralized, non-stained section revealing tiny electron-lucent globules attached to the SE hybrid layer (arrows). c: Demineralized, stained section showing a homogeneously stained SE hybrid layer. d: Higher magnification of (c) showing an SE hybrid layer with a thickness of about 1 µm. Ad: adhesive; Co: composite; De: dentin; HL: hybrid layer.

The TEM photomicrographs in Figs 5 and 6 illustrate the ultrastructure of the adhesive-dentin interfaces produced by HbMax and HbMP, respectively. Interfacial debonding during sectioning was occasionally noted for HbMax when applied in SE bonding mode, but seldom recorded for HbMP or HbMax applied in E&R bonding mode.

In general, HbMax formed thicker adhesive-resin layers compared with HbMP. An uneven distribution of nano-scale silica filler within the adhesive layer was observed for both HbMax and HbMP, with silica-filler agglomerations reaching micrometer sizes. When the adhesives were applied in E&R bonding mode, typical HAp-free hybrid layers of 4-6 μm were generated, while thinner HAp-rich hybrid layers ranging between 0.8 and 1 μm were formed in SE bonding mode. On top of and attached to the SE hybrid layer of HbMax, several round globules with low electron density were observed. These low electron-dense globules were also found within the HbMP adhesive resin close to the hybrid layer, but were smaller in size and much less frequent.

### Wsp, Wsl and Hydrophobicity/Hydrophilicity

**Fig 7:**
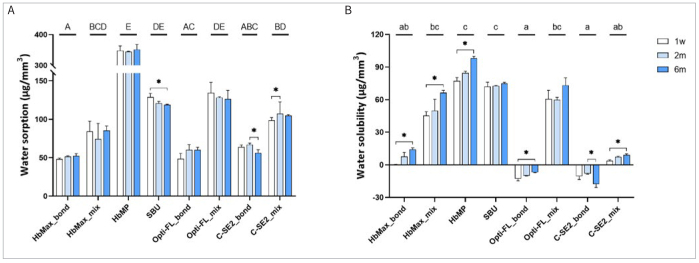
Water sorption (A) and water solubility (B) of adhesive-resin (‘bond’) disks of HbMax, HbMP, SBU, Opti-FL and C-SE2, and primer/adhesive resin mixture (‘mix’) disks of HbMax, Opti-FL and C-SE2. Groups with different capital or lowercase letters indicate significant differences in, respectively, water sorption (A) and water solubility (B) among the adhesives investigated. Bars with an asterisk indicate significant differences in water sorption (A) and water solubility (B) among the different aging periods.

**Fig 8:**
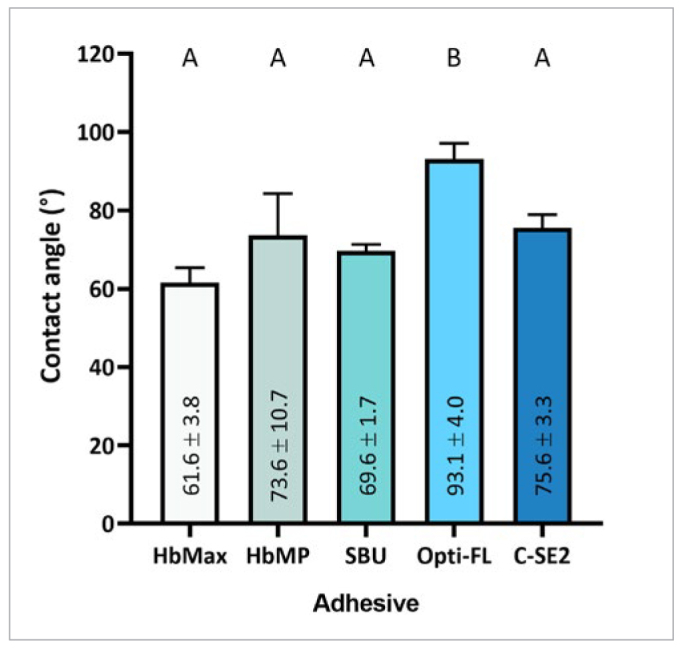
Contact-angle (CA) measurement of water applied on dentin disks treated with HbMax, HbMP, SBU, Opti-FL and C-SE2, representing their respective adhesive hydrophobicity. Groups with different capital letters indicate significant differences in CA (mean ± SD).

As shown in Table 4 and Fig 7, most water uptake occurred during the first week. Overall, the lowest water sorption (Wsp) was recorded for HbMax, while the highest was recorded for HbMP. Wsp increased after mixing the primer with the adhesive resin for all the multi-step/multi-bottle adhesives HbMax, Opti-FL, and C-SE2, with significant differences in Wsp recorded for HbMax and Opti-FL. HbMP revealed the highest water solubility (Wsl) among all the dental adhesives investigated. Mixing the primer with the adhesive resin led to an increase in Wsl, with significant differences recorded for Opti-FL. Regarding hydrophobicity/hydrophilicity (Fig 8), the water contact angle on Opti-FL-treated dentin was significantly higher than that on the dentin disks treated with other adhesives.

**Table 4:** Water sorption (Wsp) and water solubility (Wsl) of the dental adhesives investigated

Adhesive	Wsp (μg/mm^3^)^1^			Wsl (μg/mm^3^)^1^		
	1 week	2 months	6 months	1 week	2 months	6 months
HbMax_bond^2^	48.1 (1.3)	51.6 (2.4)	52.2 (3.1)	0.0 (0.5)	7.7 (1.8)	14.3 (0.7)
HbMax_mix^3^	84.2 (9.4)	74.3 (21.4)	85.5 (16.2)	45.4 (4.1)	50.0 (11.1)	66.4 (11.0)
HbMP	348.3 (25.9)	344.9 (17.2)	351.4 (14.9)	77.2 (3.8)	84.5 (1.0)	98.2 (3.4)
SBU	128.9 (4.0)	120.8 (4.2)	118.8 (1.8)	72.1 (5.0)	72.8 (0.9)	74.9 (1.4)
C-SE2_bond*	63.8 (1.7)	67.0 (1.2)	56.2 (0.1)	-10.5 (2.4)	-8.0 (1.0)	-17.7 (3.9)
C-SE2_mix	98.6 (5.1)	107.2 (8.8)	104.8 (0.8)	3.9 (1.2)	7.4 (1.9)	9.2 (0.9)
Opti-FL_bond	48.7 (2.2)	60.2 (7.2)	60.2 (3.6)	-12.7 (1.9)	-10.0 (1.6)	-7.0 (0.1)
Opti-FL_mix	134.3 (15.5)	128.1 (5.4)	126.3 (14.7)	60.6 (12.2)	59.9 (3.9)	73.3 (25.0)

^1^Medium (IQR); IQR: interquartile range; ^2^bond: adhesive resin; ^3^mix: primer-adhesive resin mixture. *Part of the data have been reported in the previous study.^40^

## Discussion

Universal adhesives (UAs) are currently popular, as they not only enable simplified, shortened application procedures but also offer the dentist a choice of bonding mode. The latter depends not only on personal preference but also according to prep-surface conditions that may favor the use of an E&R over SE bonding mode, or vice versa. For instance, young dentin in young patients is highly permeable, and may favor a less aggressive, more superficial SE bonding mode with lower risk of pulpal irritation. Highly mineralized, sclerotic, glassy, and impermeable dentin may benefit from a deeper and more aggressive E&R bonding mode, which also poses less risk of irritating the underlying pulp. However, considering that simplified 2-step E&R and 1-step SE adhesives are commonly less effective than their traditional 3-step E&R and 2-step SE adhesive precursors,^49^ respectively, similar concerns have recently been raised regarding the bonding efficacy and especially bond durability of UAs, also because long-term clinical data on UAs are still lacking.

Bis-GMA is a crosslinking monomer that has widely been used in resin-based dental materials, including dental adhesives.^17,42^ As bis-GMA is highly viscous, other co-monomers with lower viscosity, such as triethylene glycol dimethacrylate (TEG-DMA) and in particular the highly hydrophilic mono-functional monomer HEMA, are frequently added to adhesives to optimize their chemical properties.^9,46^ This beneficial effect can most likely be attributed to HEMA’s small size (low MW), which improves the diffusivity of monomers contained in dental adhesives towards and into the dental substrates.^27,28,30,45^ On the other hand, being highly hydrophilic, HEMA has been proven to increase the permeability of the adhesive-resin layer and thus the actual adhesive interface, retain more water at the interface with dentin, and promote osmotic water uptake from the deeper dentin; these are all hydrolytic bond-degradation promoting effects that can adversely affect the long-term bond durability.^29,44^ While it remains difficult to replace HEMA in adhesive formulations, manufacturers are attempting to reduce the amount of HEMA in adhesives or to replace HEMA, for instance, by acrylamide alternative monomers, which have more recently been developed,^1^ but these HEMA alternatives still require further investigation. No specific composition information of HbMax and HbMP was released by Elsodent, while this manufacturer clearly stated that both adhesives do not contain HEMA.

Apart from function, biosafety of dental materials is another factor that is raising public attention. Monomers contained in resin-based materials are known to leach once polymerized; this includes HEMA, which possesses high allergenic potential, and even bis-GMA, which contains BPA as monomer core.^38^ As an efficient cross-linker, bis-GMA is often used in resin-based materials for its mechanical property-promoting effect. BPA has been reported to be still detectable in quantifiable amounts even after long-term storage of polymerized dental composite in ethanol for up to 52 weeks, indicating that dental composites containing bis-GMA are a potential source of BPA exposure.^12^ Although this study concluded that the estimated exposure amount (1.3 ng/kg bw/day for children in a worst-case scenario) posed no health risks, this conclusion was based on the previous tolerable daily intake (TDI) of 4000 ng/kg bw/day set by the European Food Safety Authority (EFSA) in 2015. The risk of BPA has recently been re-evaluated, and an enormous decrease of TDI to 0.2 ng/kg bw/day was announced by the same authority in April, 2023.^14^ Hence, the demand for BPA-free dental materials is steadily growing, as are R&D efforts on synthesizing bis-GMA alternatives with equally efficient cross-linking potential and comparable or even better mechanical properties.^13,18,31^ Although no detailed composition information was released by Elsodent, the manufacturer claims that both the UAs HbMax and HbMP do not contain BPA.

In this study, the two HEMA- and BPA-free UAs HbMax and HbMP were evaluated and compared for bonding performance with the considered gold-standard E&R adhesive Opti-FL, the considered gold-standard SE adhesive C-SE2, and the first marketed and market-representative 1-UA SBU. HbMax is a 2-UA consisting of a separate primer and adhesive resin, while HbMP is a 1-UA combining all components in one single bottle/solution. Opti-FL and C-SE2 are considered gold-standard E&R and SE adhesives thanks to their outstanding consistent performance in numerous in-vitro laboratory studies and in-vivo long-term clinical trials,^10,33,34,36^ because of which they were employed as references/controls in this study. The 1-UA SBU has been commercially available since 2011 and has already widely been evaluated in laboratory and clinical studies; thus, it was selected to serve as market-representative UA reference.

In general, the bonding performance in terms of immediate 0TC and 50kTC-aged μTBS of the HEMA/BPA-free 2-UA HbMax and 1-UA HbMP was alike and comparable to that of the gold-standard adhesives Opti-FL and C-SE2, when these were applied in their respective manufacturer-recommended bonding mode. When compared with the reference 1-UA SBU, 2-UA HbMax as well as 1-UA HbMP significantly outperformed SBU, having revealed significantly higher immediate 0TC μTBS when applied in SE bonding mode and significantly higher 50kTC-aged μTBS when applied in E&R bonding mode. In addition, the immediate 0TC μTBS of SBU was significantly lower than that of C-SE2 when applied in SE bonding mode. Therefore, the first hypothesis tested, that the bond strength of the HEMA/BPA-free UAs to dentin would not differ from those of the reference 3-E&R, 2-SE and 1-UA adhesives, was accepted regarding the reference multi-step E&R or SE dedicated adhesives, but rejected in a positive sense regarding their superior bonding performance in respect to SBU.

To estimate bond durability, the μTBS specimens were exposed to 50k cycles of 5°-55°C thermocycling. As a commonly used laboratory method to artificially accelerate aging in vitro, thermocycling simulates a moist environment with thermal changes.^19^ Based on an estimation that such thermal changes may clinically happen 20 to 50 times per day, 10k thermal cycles are estimated to equal to 1-year service.^19^ In this respect, aging for 50k thermocycles should be regarded as a severe challenge, even more so as not the whole restored tooth but merely the sectioned μ-specimens were thermocycled with water, which was constantly in direct contact with the adhesive-dentin interface. A significant decrease in bond strength was only recorded for SBU when applied in E&R bonding mode. Although both the HEMA/BPA-free 2-UA HbMax and 1-UA HbMP revealed aging-resistant bonding performance in prediction of clinical bond durability, long-term clinical research is obviously still needed to confirm these promising laboratory data.

TEM revealed typical E&R and SE interfacial ultrastructures at dentin, corresponding to the bonding mode used for the adhesives HbMax and HbMP. The adhesive-resin layer of 2-UA HbMax was thicker than that of 1-UA HbMP, which must be related to the 2- vs 1-step application procedure. Nevertheless, this difference in adhesive-resin layer thickness did not result in a difference in bonding performance. Within the adhesive-resin layers of both HbMax and HbMP, filler clusters were relatively often observed, indicating silica-filler agglomeration. Although large agglomerations have been reported to not necessarily act as weak points,^3^ manufacturers normally attempt to avoid/eliminate filler agglomeration because they may potentially decrease the mechanical properties as well as bond strength.^22,24^ Modifying the silica-filler surface to promote more homogenous distribution in the resin matrix or advanced mixing methodologies may help to reduce such filler agglomeration.^24^ Again, this silica-filler agglomeration did not have any impact on the bonding performance of either of the UAs. More peculiar are the electron-lucent globules observed immediately adjacent and attached to the hybrid layer. These globules were detected at the adhesive-dentin interfaces produced by both adhesives, but more often and distinctly for 2-UA HbMax. Although the origin is not clear and the manufacturer Elsodent was not able to provide an explanation, these globules may represent incomplete mixing of adhesive components or phase separation, the latter typically having been documented for HEMA-free adhesives.^43,48^ HEMA acts as co-solvent to solve less water-soluble monomers in water; when HEMA is not present in a sufficiently high concentration, water, being essential in UAs to enable SE, may separate from the monomers. The fact that these interfacial globules were more often observed for HbMax than HbMP could be due to differences in composition.

The oral cavity is a wet environment where restorations and their adhesive interface with tooth tissue are continuously exposed to saliva and other dietary liquids. The ideal dental adhesive should be chemically and thermally stable without dissolution.^25^ However, the polymer network absorbs water and at the same time releases chemical components to the surrounding environment, thus affecting their mechanical and chemical properties and impairing their structure and function.^16^ In addition, water uptake and hydrolytic effects constitute the major bond-degradation mechanism. Therefore, water sorption, water solubility, and the hydrophobicity/hydrophilicity balance of dental adhesives are important parameters to be measured.

Water sorption and solubility of the pure adhesive resins and the 1:3 weight-ratio mixtures of the primer and adhesive resin combinations of the multi-step/bottle adhesives were measured after 1 week, 2 months and 6 months of water storage. Two-month water storage corresponds to the time 50k TC approximately lasts. All adhesives absorbed the most water during the first week, and this process continued over time. Among all adhesives investigated in this study, the pure HbMax adhesive resin absorbed the least water, while HbMP revealed the largest Wsp and Wsl. Although HbMax and HbMP do not contain the hydrophilic monomer HEMA, less Wsp and Wsl were not recorded for the other HEMA-containing adhesives. Dental adhesives have a complex composition, with all ingredients contributing to the final hydrophobicity/hydrophilicity balance. As expected, the 1:3 weight-ratio mixtures of the primer and adhesive resin combinations of the multi-step/bottle adhesives HbMax, C-SE2, and Opti-FL revealed higher Wsp and Wsl than did the pure adhesive-resin disks. Owing to its hydrophilic nature, partially (SE) or fully (E&R) demineralized dentin is difficult for hydrophobic monomers to infiltrate. Therefore, a primer of a multi-step/bottle dental adhesive is more hydrophilic, since it contains more solvents (including water) and more hydrophilic monomers. As a bond promoter, a primer’s function is to improve surface wetting and prepare the dentin surface for effective infiltration of the more hydrophobic monomers contained in the adhesive resin.^7,49^ Ultimately, the adhesive should bridge the hydrophilic dentin to the hydrophobic restorative composite and transform the adhesive interface into a state as hydrophobic as possible, thus to prevent water uptake and hydrolytic bond-degradation effects. Overall, the second hypothesis tested in this study, that water sorption and solubility of the HEMA/BPA-free 2-UA HbMax and 1-UA HbMP would not differ from those of the reference 3-E&R, 2-SE and 1-UA adhesives, was partially rejected when only pure adhesive resins were compared, as the 1-step UAs significantly absorbed more water than did the adhesive resins of the 3-E&R, 2-SE, and 2-UA adhesives.

The water solubility of Opti-FL and C-SE2 was negative, indicating the resin disks gained weight upon water storage, a phenomenon that was also reported in previous studies.^25,51^ Upon water immersion of a resin disk, the polymer matrix releases uncured monomers entrapped in the network and simultaneously interacts with absorbed water to form “bound water” via hydrogen binding.^32^ The negative Wsl data recorded for Opti-FL and C-SE2 do not mean that the resin disks were insoluble. The recorded negative Wsl is the net effect of weight loss by dissolution and weight gain by the formation of bound water. If bound water formation exceeds monomer elution, the net weight of the resin disks increases upon water storage, by which a negative water solubility is recorded. However, such negative Wsl data were only recorded for the pure adhesive resins of the multi-step E&R and SE adhesives. For primer/adhesive resin mixtures, water solubility was always positive, indicating monomers eluted more upon immersion in water than water was bound into the polymer matrix. Besides differences in hydrophilicity, Wsl also depends on the degree of polymerization conversion, e.g., with primer/adhesive resin mixtures proportionally possessing fewer photo-initiators.

Apart from water sorption and solubility, the hydrophobicity of adhesive-treated dentin was measured by measuring the contact angle (CA) of a water droplet on the surface. Among all adhesives investigated, only Opti-FL presented a significantly higher CA, even exceeding 90 degrees. The third hypothesis tested in this study, that the hydrophobicity of the HEMA/BPA-free 2-UA HbMax and 1-UA HbMP would not differ from that of the reference 3-E&R, 2-SE, and 1-UA adhesives, was accepted except when compared to Opti-FL. Normally, a 90-degree CA is regarded as the boundary between a hydrophilic and hydrophobic state.^21,23^ No clear relationship between the Wsp and CA results was found.

## Conclusion

Having 1. measured the bonding effectiveness in terms of μTBS of the HEMA- and BPA-free 2-UA HbMax and the HEMA- and BPA-free 1-UA HbMP to (flat) dentin when applied in both E&R and SE bonding modes, 2. characterized their respective interfacial ultrastructure at dentin by TEM, and 3. additionally measured their water sorption, water solubility, and hydrophobicity/hydrophilicity, HbMax and HbMP revealed bonding performance similar to that of the gold-standard E&R and SE dental adhesives, despite differences in water sorption/solubility and hydrophobicity/hydrophilicity.

## Clinical relevance:

The HEMA/BPA-free UAs can achieve durable bonding to flat dentin comparable to that of the E&R and SE dental adhesives.
